# Active and Passive Immunization with Myelin Basic Protein as a Method for Early Treatment of Traumatic Spinal Cord Injury; a Meta-Analysis 

**DOI:** 10.22037/aaem.v9i1.1316

**Published:** 2021-08-30

**Authors:** Mahmoud Yousefifard, Arian Madani Neishaboori, Seyedeh Niloufar Rafiei Alavi, Amirmohammad Toloui, Mohammed I M Gubari, Amirali Zareie Shab Khaneh, Maryam Karimi Ghahfarokhi, Mostafa Hosseini

**Affiliations:** 1 Physiology Research Center, Iran University of Medical Sciences, Tehran, Iran.; 2 Community Medicine, College of Medicine, University of Sulaimani, Sulaimani, Iraq.; 3 Department of Epidemiology and Biostatistics, School of Public Health, Tehran University of Medical Sciences, Tehran, Iran.; 4 Sina Trauma and Surgery Research Center, Tehran University of Medical Sciences, Tehran, Iran.; a First and second authors have equally contributed to this work

**Keywords:** Early Medical Intervention, Emergency treatment, Immunization, Myelin basic protein, Spinal cord injuries

## Abstract

**Introduction:**

Traumatic spinal cord injury (SCI), as a dangerous central nervous system damage, continues to threaten communities by imposing various disabilities and costs. Early adjustment of the immune system response using Myelin Basic Protein (MBP) immunization may prevent the SCI-related secondary damages. As a result, the current study is designed to review and analyse the evidence on active and passive immunization with MBP for treatment of traumatic SCI.

**Methods:**

Medline, Embase, Scopus, and Web of Science databases were systematically searched until the end of 2020. Criteria for inclusion in the current study included pre-clinical studies, which performed passive (injection of MBP-activated T cells) or active (administration of MBP or MBP-modified peptides) immunization with MBP after traumatic SCI. Exclusion criteria was defined as lack of a non-treated SCI group, lack of evaluation of locomotion, review studies, and combination therapy. Finally, analyses were conducted using STATA software, and a standardized mean difference (SMD) with a 95% confidence interval (CI) were reported.

**Results:**

Data from 17 papers were included in the present study. Finally, analysis of these data showed that passive immunization (SMD=0.87; 95%CI: 0.19-1.55; p=0.012) and active immunization (SMD=2.08, 95%CI: 1.42-2.73; p<0.001) for/with MBP both have good efficacy in improving locomotion following traumatic SCI. However, significant heterogeneity was observed in both of them. The most important sources of heterogeneity in active immunization were differences in SCI models, route of administration, time interval between SCI and transplantation, and type of vaccine used. In passive immunization, however, these sources were the model of SCI and the time interval between SCI and transplantation. Although, there was substantial heterogeneity among studies, subgroup analysis showed that active immunization improved locomotion after traumatic SCI in all tested conditions (with differences in injury model, severity of injury, method of administration, different time interval between SCI to vaccination, etc.).

**Conclusion:**

The results of the present study demonstrated that immunization with MBP, especially in its active form, could significantly improve motor function following SCI in rats and mice. Therefore, it could be considered as a potential treatment in acute settings such as emergency departments. However, the safety of this method is still under debate. Therefore, it is recommended for future research to focus on the investigation of safety of MBP immunization in animal studies, before conducting human clinical trials.

## 1. Introduction:

Spinal cord injury (SCI) is one of the most dangerous neurological disorders that mostly affects the young population, and causes long-term disabilities in this group of society. Unfortunately, more than 90% of the patients suffer from long-term motor disabilities and about 78% of them experience severe to moderate pain. SCI and its complications impose significant direct and indirect costs on both the individual and the healthcare system; the annual costs of SCI are estimated at about 26270$ per patient (1).

Current treatments have very low efficacy and can only alleviate some of its symptoms. Medication therapy is the mainstay of current treatment methods. Not only has this treatment had very little effect on motor recovery (2), but also the unwanted side effects that occur with continued use of medications are a major barrier to their use (3). Current efforts in improving the recovery of central nervous system (CNS) focus on two aspects: 1) stimulation of neurogenesis or regeneration and 2) neuroprotection or prevention of secondary damage (4, 5). However, there is still significant disagreement over the new treatment strategies. For instance, older studies had demonstrated that inflammation caused by autoimmune response leads to exacerbation of SCI and motor impairment (6, 7). Nonetheless, recent animal research showed that the post-injury autoimmune reactions provoke beneficial endogenous responses following SCI (8). These studies indicate that the presence of immune T cells in the injury site increases secretion of nervous growth factors, improves the tissue environment surrounding the damaged neurons, protects the remaining myelin, and eventually, leads to enhancement of motor recovery (4).

It has also been demonstrated that proper regulation of immune response following SCI may have an essential role in axonal regeneration, prevention of secondary injuries, and SCI recovery. In addition, local transplantation of macrophages was associated with some degree of motor recovery in the literature (9, 10).

Myelin Basic Protein (MBP) is a surface antigen expressed on cells in the CNS. Available studies show that transplantation of activated immune cells against this antigen is associated with improved motor recovery in animals with SCI (11, 12). Thus, transplantation of cells activated by this antigen, may selectively affect the CNS and reduce nonspecific inflammatory responses in other tissues (13). On the other hand, administration of this protein or similar peptides, as an active immunogenic process, could have significant effects on improving motor function following SCI (14-16). 

Although several studies that evaluate the effectiveness of active and passive immunization in SCI are available, conflicting findings have been reported between studies. For example, Rodríguez-Barrera et al. showed that active immunization with neural-derived peptides, such as MBP-related peptides, improves motor function following SCI (17). However, Ibara et al. showed that the use of this immunization has no effect on motor function following SCI (18). These inconsistencies have made it impossible to draw a general conclusion in this regard. On the other hand, it is not yet clear which active and inactive immunogenic methods are more effective in improving the motor function of animals.

Accordingly, there is still no conclusion on the role of passive immunization with immune cells activated by MBP or on active immunization with injection of MBP and its derivatives in the treatment of SCI in the literature. Hence, the present study aims to perform a systematic review and meta-analysis to determine the effectiveness of passive or active immunization with MBP on motor recovery in animal models following SCI.

## 2. Methods:

**2.1. Study design**

PICO was defined as follows: Problem (P) included animals in which SCI injury was induced. Intervention (I) was the injection of MBP-activated T cells (passive immunization) or administration of MBP or MBP-modified peptides, inducing intrinsic immunity to MBP (active immunization). Comparison (C) consisted of a comparison with control animals, which were induced with SCI but had not received treatment, and outcome (O) was an assessment of motor function by BBB testing.

2.2. Search strategy

An extensive search was conducted on Medline, Embase, Scopus, and Web of Science databases until the end of 2020. Search strategy was designed based on keywords related to SCI and immunization/vaccination, which were obtained through searching databases such as Emtree (Embase) and Mesh (PubMed). 

Although only animal studies were included in the current systematic review, animal study filter was not applied in the search, in order to prevent the loss of relevant records. A manual search was also performed in the list of references of the relevant articles and highly related journals. To search for Grey literature, the thesis section was searched in the ProQuest database. In addition, Google and Google Scholar search engines were searched to find additional resources. Finally, authors of the relevant studies were contacted to gain access to their unpublished or pre-print data. Full electronic search strategy for all databases is presented in appendix 1. 

**2.3. Selection criteria**

The inclusion criteria in the present study consisted of controlled studies that had evaluated the efficacy of immune cells activated against MBP protein in motor function improvement after SCI. Controlled studies were defined as studies that had a control group without any treatment (placebo group or vehicle group), in addition to the group treated with active/passive immunization with MBP. Since some articles were written in Chinese and Japanese, no language restrictions were imposed. Irrelevant, duplicate and review studies were excluded. Moreover, lack of motor function evaluation and combination therapy were the other exclusion criteria in the current meta-analysis.

**2.4. Data collection and quality assessment**

The search results in the databases were combined and duplicate studies were eliminated using Endnote software. Next, after screening of the titles and abstracts of the records by two independent reviewers, full texts of potentially relevant studies were obtained and then relevant studies were included. The final results of the systematic search in the present study were recorded in a checklist designed based on PRISMA statement (19). The extracted data consisted of study design, treated and control group characteristics (age, sex, spinal cord injury model, etc.), sample size, outcome (motor outcome) and possible biases. Considering that motor function recovery following SCI needs a 4-week time window after the injury in a rat model, studies with a follow-up period of less than four weeks were excluded. In cases that the aforementioned information was not reported, the corresponding author of the study was contacted and asked to provide the required data. If the results were presented in the form of graphs, they were extracted using the method demonstrated by Sistorm and Mergo (20). In cases of disagreement between the two researchers, it would be resolved through discussion with a third reviewer. Quality assessment of the studies was conducted independently by the two researchers based on the recommended SYRCLE guideline (21). This tool includes 10 domains of sequence generation, baseline characteristics, allocation concealment, random housing, care-giver blinding, random outcome assessment, blinding of outcome assessor, incomplete outcome assessment, selective outcome assessment, and other risk of bias. Two independent reviewers rated each domain (as low risk, high risk, unclear) according to signaling questions presented in explanation and elaboration paper of SYRCLE guideline (21). Any disagreement was resolved by discussion.

**2.5. Statistical analysis**

In all of the studies, the scores of the animals in the BBB test in treated and control groups were considered as the final outcome. Data were recorded as mean and standard deviation and analyzed using “metan” command in STATA 14.0 statistical software. The findings were reported as standardized mean difference (SMD) and 95% confidence interval (95% CI). Heterogeneity between the studies was assessed using the I^2^ test and a p value of less than 0.1 was considered as significant heterogeneity. If the studies were homogeneous, the fixed effect model was performed, and in case of heterogeneity, subgroup analysis was conducted to determine the source of the heterogeneity. Random effect model was performed in cases where the cause of heterogeneity was not clear. Meta-analyses were performed, only if the data were reported in at least two separate experiments. Finally, publication bias was investigated by performing Egger’s test and presenting a funnel Plot (22).

## 3. Results:

**3.1. Study characteristics**

**Figure 1:**
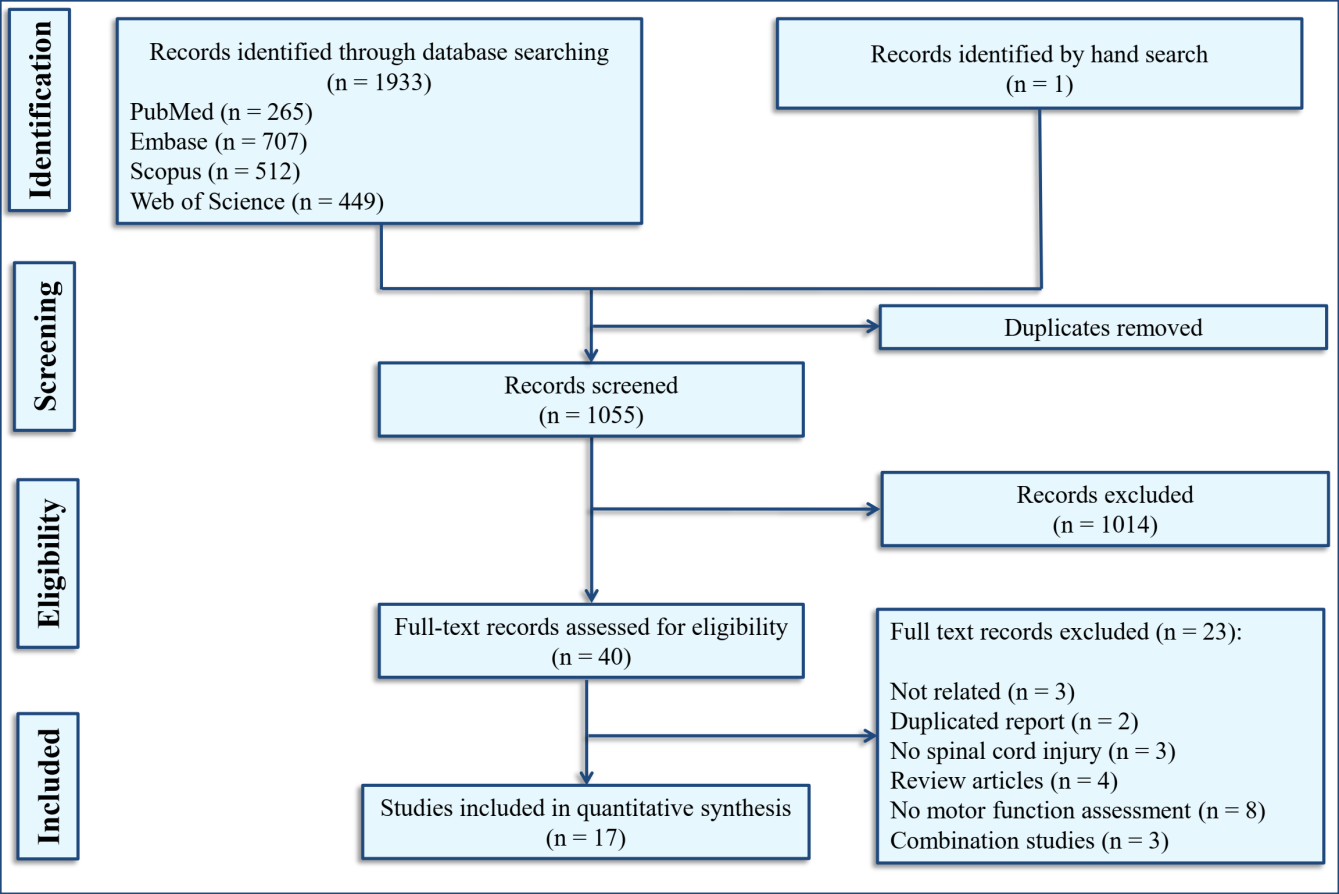
Flowchart of selecting related studies

Searching the databases eventually provided researchers with 1055 non-duplicate records. By reviewing the abstracts and titles of the records, the full text of 40 relevant articles were obtained and then studied in detail. Finally, the data of 17 articles (5, 11, 12, 14-18, 23-30) were included in the present meta-analysis (Figure 1). These pre-clinical articles consisted of 39 separate analyses (experiments), 36 of which were performed on rats and 3 were performed on mice. The animals were female in 38 of the experiments and male in only one. The models for the induction of SCI were contusion in 31, compression in four, hemisection in one and transection model in three experiments. The severity of injury was moderate in 22 and severe in 17 of the trials. The site of injury was thoracic in all of the experiments. Seven experiments had used prophylaxis vaccination and 32 of them performed the vaccination after the SCI. 25 experiments vaccinated the subjects immediately after the SCI. 13 experiments conducted passive immunization by injecting MBP-activated T cells, while 26 experiments performed active immunization. Compounds used to induce active immunization included MBP, A91 (a peptide derived from MBP), and dendritic cells pulsed with MBP or A91. Moreover, follow-up time ranged from 27 days to 112 days (Table 1).

**3.2. Efficacy of passive immunization on motor function recovery after SCI**

**Figure 2:**
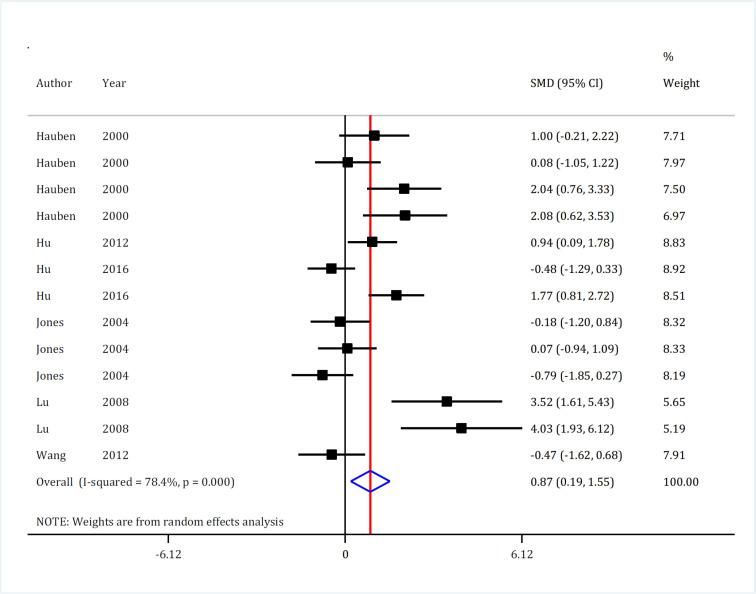
Forest plot for effect of passive immunization with Myelin Basic Protein (MBP) on motor function recovery after spinal cord injury. SMD: Standardized mean difference; CI: Confidence interval

The effect of passive immunization on improving the motor function of animals following SCI was investigated in 13 experiments. Meta-analysis showed that passive immunization improved motor function of animals after SCI (SMD = 0.87; 95% CI: 0.19, 1.55; p = 0.012). Significant heterogeneity was observed in this section (I^2^ = 78.4%; p <0.001) (Figure 2). Therefore, subgroup analysis was conducted. The most important sources of heterogeneity and subgroup analysis results are reported in Table 2. The model of injury and the time interval between SCI and transplantation were the most important sources of heterogeneity. Studies that used transection/hemisection models (I^2^ = 0.0%) and had a time interval of 1-9 days between SCI and transplantation (I^2^ = 49.9%) were found to be homogeneous. 

Interestingly, the efficacy of passive immunization on motor recovery was affected by the diversities between the studies. Passive immunization improved motor function only in models of contusion injury (SMD = 1.23, p <0.001), whereas such an effect was not observed in transection/hemisection models (SMD = -0.19; p = 0.565). Also, the use of passive immunization in severe SCI models had no effect on motor function of the animals (SMD = 0.47, p = 0.262), but in moderate injuries, it significantly enhanced motor function (SMD = 1.30, p = 0.018). In addition, intraperitoneal injection of MBP-activated T cells (SMD = 0.54, p = 0.173) and the administration of this treatment immediately after SCI (SMD = 0.77, p = 0.052) had no effect on the motor function of animals. Finally, the impression is created that the positive effects of passive immunization is only observed in long term follow-up (p = 0.024) (Table 2).

**3.3. Efficacy of active immunization on motor function recovery after SCI**

**Figure 3:**
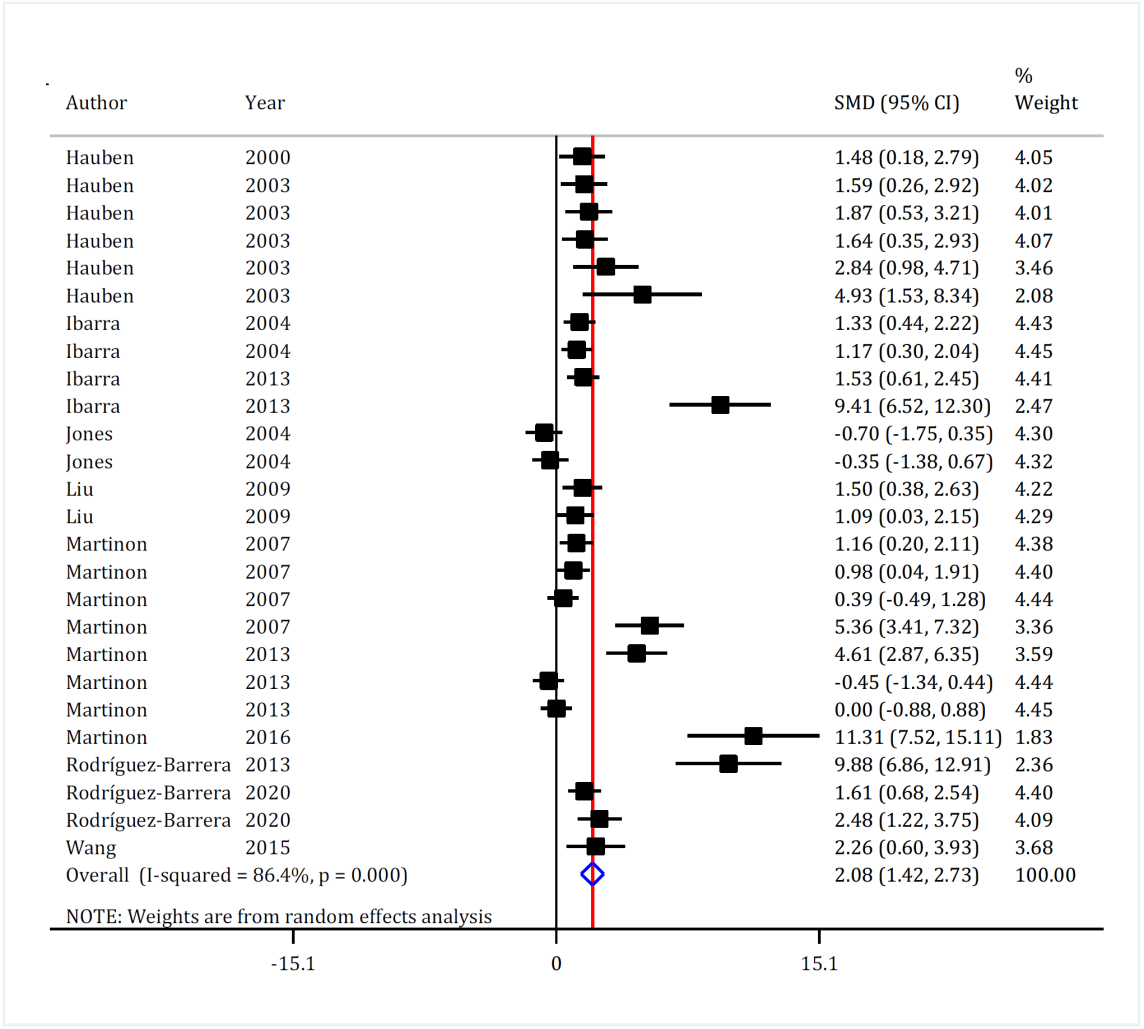
Forest plot for effect of active immunization with Myelin Basic Protein (MBP) on motor function recovery after spinal cord injury. SMD: Standardized mean difference; CI: Confidence interval

The effect of active immunization on the motor function of the animals was evaluated in 26 experiments. Meta-analysis of this section demonstrated that active immunization significantly improves motor function following SCI (SMD = 2.08, 95% CI: 1.42, 2.73; p <0.001) (Figure 3). Significant heterogeneity was observed in this part (I^2^ =86.4%, p <0.001). Subgroup analysis showed that differences in SCI model, route of administration, time interval between SCI and transplantation, and the type of vaccine inducing active immunization were the most important sources of heterogeneity. Studies that had used compression model to induce SCI (I^2^ = 0.0%), studies with intraperitoneal (I^2^ = 26.9%) or intrathecal administration (I^2^ = 0.0%), studies that performed immunization in the chronic phase (60 days after SCI) (I^2^ =16.8%), and studies that had used activated dendritic cells to create immunization (I^2^ = 0.0%) were homogeneous. It is worth mentioning that active immunization improved the motor function in the animals in all test conditions (with differences in injury model, severity of injury, method of administration, etc.) (Table 3).

**3.4. Publication bias and risk of bias assessment**

**Figure 4:**
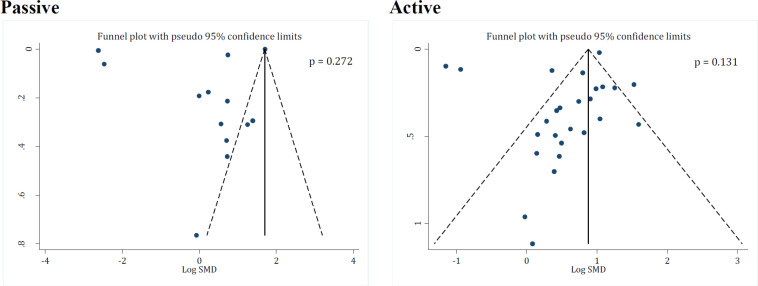
Funnel plot for assessment of publication bias in studies assessing the effect of passive and active immunization with Myelin Basic Protein (MBP) on motor function recovery after spinal cord injury. SMD: Standardized mean difference

The SYRACLE tool was used to assess the risk of bias in the included studies. Accordingly, all of the studies in this review had low risk of bias in the baseline characteristic section. On the other hand, all of the articles had an unclear risk of bias on random housing. In both care giver blinding and allocation concealment items, there was one study with unclear risk of bias, three had low risk of bias, and the others were classified as having high risk of bias. However, only two studies had high risk of bias in the blinding of outcome assessor section and the others were classified as having low risk of bias. More information on the risk of bias of the articles is shown in Table 4. It should be kept in mind that publication bias was not observed in either active immunization (p = 0.131) or passive immunization studies (p = 0.272) (Figure 4). 

## 4. Discussion:

The present study aimed to determine the efficacy of passive and active immunization with MBP on motor function recovery following SCI in animal models. The obtained results showed that overall, both passive and active immunization with MBP can improve motor function recovery following SCI in rats and mice. This substantiates previous findings in the literature indicating the positive effects of controlled activity of the immune system in the injury site. In other words, the autoimmunity that occurs due to the activation of immune cells, (including T-helper 2, macrophages, and neutrophils), protects the spinal cord from further damage, whilst promoting tissue recovery (10, 11, 31).

Based on the results of the current review, in general, active immunization has a greater effect on the protection and recovery of the spinal cord following injury, compared to passive immunization. This finding is very promising, as storage of antigens in the laboratory and the clinic is much more attainable than activated immune cells. Furthermore, injection of antigens is associated with less complications and side effects. Previous studies have also noted the pathogenic effect of most injected T cells on the central nervous system (32). In addition, the formation of memory cells in active immunization provides a more durable immunity and it is expected to protect the injured spinal cord from further damage for a longer time. It has even been observed that in chronic conditions (60 days after injury) the cells formed under the influence of active immunization could maintain their function and lead to recovery after spinal cord injury (17).

Subgroup analysis showed that passive immunization is not effective in promoting motor recovery after SCI in severe or immediate administration after the injury. Hence, their administration is not recommended in such conditions. In contrast, active immunization is less associated with the acuity of the injury and has significant efficacy in all conditions. This statement could be justified considering the vulnerability of cells to antigens (10). It may even be suggested that in situations in which immunization is achieved by the injection of dendritic cells, the immunity is safer and more long lasting, granted that the immune cells become activated inside the body of the animal itself (12, 23, 26).

At last, it should be added that immunization, and especially active immunization, have shown favorable efficacy in animal studies. Further investigations are recommended to focus on the application of this method both for the treatment of SCI (23, 25, 27-30) and also prophylaxis (vaccination) (14, 15, 18) in high-risk groups, e.g. horse riders or rally drivers. However, the safety of this method is still under debate, as autoimmune diseases, such as multiple sclerosis, are caused by hyperreaction to intrinsic antigens, such as MBP (33). This issue may also be considered as one of the limitations of the present study, as none of the included studies had discussed it. However, there are studies that demonstrate the beneficial effects observed when administrating T Cell vaccines in Multiple Sclerosis patients (34). Hence, investigations on the safety of active/passive vaccination with MBP are strongly suggested to be carried out before conducting clinical trials. 

Moreover, another limitation of this study was the heterogeneity of the articles. Considering the sources of this heterogeneity, including the method of injury, the time interval between SCI and treatment, and the route of administration, it is proposed that further research should be undertaken regarding the mentioned variables. Finally, due to the mentioned limitations, conclusions about the application of active and passive immunization in SCI should be drawn with caution.

**Appendix 1:** Search syntaxes for current study

**PubMed**
((Immunization[Mesh] OR Active Immunization*[Mesh] OR Passive Immunization*[Mesh] OR Vaccination[Mesh] OR Autoimmunity[Mesh] OR Immunization, Passive/methods[Mesh] OR Immunization[tiab] OR Active Immunization*[tiab] OR Passive Immunization*[tiab] OR Vaccination[tiab] OR Autoimmunity[tiab] OR Immunization, Passive/methods[tiab] OR Autoimmunities[tiab] OR autoimmune Response*[tiab] OR Myelin Basic Protein[tiab] OR Immunization with neural derived peptides[tiab] OR A91[tiab])) AND (Spinal cord injuries[mh] OR Spinal cord contusion[tiab] OR Spinal cord transection[tiab] OR Injured spinal cord[tiab] OR spinal cord Traum*[tiab] OR Spinal cord Hemisection[tiab] OR Spinal compression[tiab] OR traumatic Myelopath*[tiab] OR spinal cord Laceratio*[tiab] OR post-traumatic Myelopath*[tiab])
**Embase**
1. 'active immunization'/exp OR 'passive immunization'/exp OR 'vaccination'/exp OR 'myelin basic protein'/exp 2. 'spinal cord injury'/exp OR 'experimental spinal cord injury'/exp OR 'spinal cord transsection'/exp OR 'cervical spinal cord injury'/exp OR 'cervical spinal cord injury'/exp OR 'cervical spinal cord injury'/exp OR 'lumbar spinal cord'/exp OR 'photochemical spinal cord injury'/exp OR 'spinal paralysis'/exp OR 'spinal cord transverse lesion'/exp 3. #1 AND #2
**Scopus**
( ( TITLE-ABS-KEY ( "active immunization" ) OR TITLE-ABS-KEY ( "passive immunization" ) OR TITLE-ABS-KEY ( "vaccination" ) OR TITLE-ABS-KEY ( "myelin basic protein" ) OR TITLE-ABS-KEY ( "Myelin Basic Protein" ) OR TITLE-ABS-KEY ( "Immunization with neural derived peptides" ) OR TITLE-ABS-KEY ( "A91" ) ) ) AND ( ( ( TITLE-ABS-KEY ( "spinal cord injuries" ) OR TITLE-ABS-KEY ( "spinal cord injury" ) OR TITLE-ABS-KEY ( "spinal cord transection" ) OR TITLE-ABS-KEY ( "spinal cord hemisection" ) OR TITLE-ABS-KEY ( "injured spinal cord" ) OR TITLE-ABS-KEY ( "spinal cord trauma" ) OR TITLE-ABS-KEY ( "spinal compression" ) OR TITLE-ABS-KEY ( "spinal cord contusion" ) OR TITLE-ABS-KEY ( "photochemical spinal cord injury" ) OR TITLE-ABS-KEY ( "spinal paralysis" ) ) ) )
**Web of Science**
((TS="active immunization" OR "passive immunization" OR "vaccination" OR "myelin basic protein" OR "Myelin Basic Protein" OR "Immunization with neural derived peptides" OR "A91") AND (TS="spinal cord injuries" OR "spinal cord injury" OR "spinal cord transection" OR "spinal cord hemisection" OR "injured spinal cord" OR "spinal cord trauma" OR "spinal compression" OR "spinal cord contusion" OR "photochemical spinal cord injury" OR "spinal paralysis") )

**Table 1:** Characteristics of included studies

**Study**	**Sex, Strain, Species**	**Severity of injury; Model; Injury Location**	Injury to treatment* (day)	**Vaccine**	**Number of administrations**	**Type of Treatment**	**Transplantation route**	**Number of cells**	**Follow-up days**
Hauben, 2000 (14)	Female, Lewis, Rat	Severe; Contusion, transection; T9, T7	-7, 0, 7	T_MBP_ cells, MBP-IFA	1	Passive, active	IP, SC	1.0 × 10^7^	76
Hauben, 2003 (23)	Female, Lewis, Rat	Severe; Contusion; T8	0	DC-MBP, DC-A91	1	Active	IS, IV, SC	5.0 × 10^5^ 1.0 × 10^7^ 2.0 × 10^7^	76
Hu, 2012 (24)	Female, Lewis, Rat	Moderate; Contusion; T9	9	T_MBP_ cells	1	Passive	IV	4.0 × 10^5^	49
Hu, 2016 (25)	Female, Lewis, Rat	Moderate; Contusion; T9	0	T-helper 1 _MBP_ cell, T-helper 2 _MBP_ cell	1	Passive	IV	2.0 × 10^7^	42
Ibarra, 2004 (18)	Female, Lewis and SD, Rat	Severe; Contusion; T9	-7	A91-CFA	1	Active	SC	NA	72
Ibarra, 2013 (15)	Female, SD, Rat	Moderate; Contusion; T9	-40	A91-CFA	1, 2	Active	SC	NA	63
Jones, 2004 (5)	Female, Lewis, Rat	Moderate, Severe; Contusion, Transection; T8	-7, 0	T_MBP_ cells, MBP-IFA, MBP-CFA	1	Passive, active	IP, SC	1.0 × 10^7^	43, 63
Liu, 2009 (26)	NR, BALB/C, Mice	Moderate; Compression; T10	1	DC-MBP	1	Active	IP, IS	5.0 × 10^5^	84
Lu, 2008 (27)	Female and Male, SD, Rat	Moderate; Contusion; T9	0	T_MBP_ cells	1	Passive	IV	2.0 × 10^7^	56
Martinon, 2007 (28)	Female, SD, Rat	Moderate; Contusion, compression; T9	0	A91-CFA	1, 2	Active	SC	NA	77, 84
Martinon, 2013 (29)	Female, SD, Rat	Moderate, severe; Contusion, transection; T9	0	A91-CFA	1	Active	SC	NA	56
Martinon, 2016 (30)	Female, SD, Rat	Moderate; Contusion; T9	0	A91-CFA	1	Active	SC	NA	112
Rodríguez-Barrera, 2013 (16)	Female, SD, Rat	Moderate; Contusion; T9	0	A91-CFA	1	Active	SC	NA	30
Rodríguez-Barrera, 2020a (17)	Female, SD, Rat	Moderate; Contusion; T9	60	A91	2	Active	SC	NA	60
Rodríguez-Barrera, 2020b (17)	Female, SD, Rat	Moderate; Contusion; T9	60	A91-CFA	1	Active	SC	NA	60
Wang, 2012 (11)	Female, SD, Rat	Severe; Transection; T9	0	T_MBP_ cells	1	Passive	IV	2.0 × 10^7^	56
Wang, 2015 (12)	NR, BALB/C, Mice	Moderate; Compression; T9	1	DC-A91	1	Active	IP	1.0 × 10^6^	27

CFA: Complete Freund’s adjuvant; DC: Dendritic cells; DC-MBP: DCs pulsed with myelin basic protein; DC-A91: DCs pulsed with A91 peptide; IFA: Incomplete Freund’s adjuvant; IP: Intraperitoneal; IS: Intraspinal; IV: Intravenous; MBP: Myelin basic protein; NA: Not applicable; SC: Subcutaneous; SD: Sprague-Dawley; T: Thoracic regions; T_MBP_: Myelin basic protein-activated T cells; MBP-IFA: MBP emulsified in incomplete Freund’s adjuvant; MBP-CFA: MBP emulsified in complete Freund’s adjuvant.

*, Negative numbers refer to pre-treatment protocols.

**Table 2:** Subgroup analysis for effect of passive immunization with Myelin Basic Protein (MBP) on motor function recovery after spinal cord injury (SCI)

**Variable**	**No. experiment**	**Heterogeneity (p value)**	**SMD (95% CI)**	**P**
**Model of injury**				
Contusion	10	81.1% (<0.001)	1.23 (0.39, 2.07)	**<0.001**
Transection/hemisection	3	0.0% (0.796)	-0.19 (-0.81, 0.45)	0.565
**Severity of injury**				
Moderate	6	84.6% (<0.001)	1.39 (0.24, 2.55)	**0.018**
Severe	7	71.3% (0.002)	0.47 (-0.35, 1.30)	0.262
**Route of administration**				
Intraperitoneal	7	68.7% (0.004)	0.54 (-0.24, 1.32)	0.173
Intravenous	6	85.7% (<0.001)	1.33 (0.10, 2.56)	**0.034**
**Time interval between SCI and transplantation**				
Immediately after SCI	11	79.6% (<0.001)	0.77 (-0.006, 1.55)	0.052
1-9 days after SCI	2	49.9% (0.158)	1.38 (0.32, 2,44)	**0.011**
**Follow-up duration**				
< 8 weeks	4	80.0% (0.002)	0.51 (-0.50, 1.52)	0.327
≥ 8 weeks	15	80.1% (<0.001)	1.11 (0.14, 2.07)	**0.024**

CI: confidence interval; SMD: Standardized mean difference.

**Table 3:** Subgroup analysis for effect of active immunization with Myelin Basic Protein (MBP) on motor function recovery after spinal cord injury (SCI)

**Variable**	**No. experiment**	**Heterogeneity (p value)**	**SMD (95% CI)**	**P**
**Model of injury**				
Contusion	21	88.5% (<0.001)	2.42 (1.61, 3.23)	**<0.001**
Compression	4	0.0% (0.567)	1.28 (0.72, 1.84)	**<0.001**
Transection	1	NA	NA	NA
**Severity of injury**				
Moderate	16	90.1% (<0.001)	2.67 (1.68, 3.68)	**<0.001**
Severe	10	68.9% (0.001)	1.27 (0.59, 1.95)	**<0.001**
**Route of administration**				
Intraperitoneal	2	26.9% (0.242)	1.49 (0.40, 2.90)	**0.008**
Intravenous	1	NA	NA	**NA**
Subcutaneous	19	89.8% (<0.001)	2.29 (1.44, 3.14)	**<0.001**
Intraspinal	4	0.0% (0.981)	1.64 (1.01, 2.27)	**<0.001**
**Time interval between SCI and transplantation**				
Prophylaxis	7	88.4% (<0.001)	1.50 (0.28, 2.71)	**0.016**
Immediately after SCI	17	87.3% (<0.001)	2.42 (1.52, 3.31)	**<0.001**
60 days after SCI	2	16.8% (0.273)	1.94 (1.10, 2,77)	**<0.001**
**Type of vaccine**				
MBP or A91 base antigens	18	90.1% (<0.001)	1.11 (0.85, 1.36)	**<0.001**
Activated dendritic cells	8	0.0% (0.446)	1.73 (1.23, 2.22)	**<0.001**
**Follow-up duration**				
< 8 weeks	4	81.1% (<0.001)	5.12 (0.50, 9.73)	**0.030**
≥ 8 weeks	21	95.8% (<0.001)	1.67 (1.10, 2.24)	**<0.001**

CI: confidence interval; SMD: Standardized mean difference.

**Table 4:** Risk of bias assessment of included studies

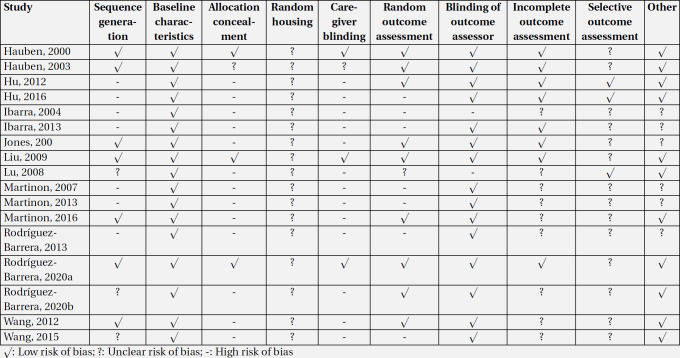

## 5. Conclusion

Although the heterogeneity among the included studies in the present meta-analysis was significant, the result of this study showed that the immunization provided with MBP, especially in its active form, significantly improves motor function following SCI in rats and mice. However, future investigations are necessary in order to establish the efficacy of this method. In addition, safety of immunization with MBP is debated, both in active and passive immunization. Hence, considering the possible complications of this method, such as autoimmunity, it is recommended for future researchers to investigate its safety by designing more animal experiments, before conducting clinical trials. 

## 6. Declaration:

### 6.1. Acknowledgment

None.

### 6.2. Funding

This study has been funded and supported by Sina Trauma and Surgery Research Center, Tehran University of Medical Sciences, Tehran, Iran; Grant No: 95-01-38-31334

### 6.3. Author contribution

Study design: MH, MY; Data gathering: MY, AMN, SNRA, AT; Analysis: MH, AZSK, and MKG MY; interpreting the results: All authors; Drafting: MY, AMN; Critically revised: All authors.

### 64. Conflict of interest

All authors declare that there is no conflict of interest. 
